# Long-term, real-world experience of pasireotide dose reduction in patients with acromegaly

**DOI:** 10.1530/EC-23-0155

**Published:** 2023-09-13

**Authors:** Nelma Veronica Marques, Luiz Eduardo Armondi Wildemberg, Monica R Gadelha

**Affiliations:** 1 Neuroendocrinology Research Center, Endocrinology Section, Medical School and Hospital Universitário Clementino Fraga Filho, Universidade Federal do Rio de Janeiro, Rio de Janeiro, Brazil

**Keywords:** acromegaly, pasireotide, long-term, hyperglycaemia, dose reduction

## Abstract

Pasireotide long-acting release is effective in achieving biochemical control and reducing tumour volume in patients with acromegaly inadequately controlled by first-line therapy. As part of a long-term, real-world study at our centre, 20 of 50 patients receiving pasireotide benefited from a reduction in pasireotide dose. Pasireotide reduced insulin-like growth factor 1 (IGF1) levels to below the upper limit of the normal range, with some patients responding within 1−3 months of treatment (*n* = 11) and others after ≥4 months (*n* = 9). Following pasireotide dose reduction, IGF1 levels showed a mild increase but remained within the normal range after a median of 39 months in the early responders and 17 months in the late responders. Glucose and glycated haemoglobin levels decreased following dose reduction. Identifying patients who may benefit from a reduction in pasireotide dose warrants further research as it may improve the management of pasireotide-associated hyperglycaemia in susceptible patients.

**Significance statement:**

Patients with acromegaly often need medical therapy for extended periods of time, and pasireotide is an effective, long-term treatment option. However, pasireotide may increase blood glucose levels in some patients, such as those with pre-existing diabetes. In this single-centre study, we show that following dose reduction of pasireotide over time, patients with acromegaly maintained their biochemical response (IGF1 < ULN) and had improved glycaemic control. As such, dose reductions may be an effective, personalised treatment approach for managing some patients receiving long-term pasireotide therapy and could allow patients to achieve early and long-term biochemical control while minimising adverse drug effects.

## Introduction

Pasireotide long-acting release is a multireceptor-targeted somatostatin receptor ligand (SRL) approved to treat adult patients with acromegaly (1). In patients, this second-generation SRL has been shown to be effective in normalising insulin-like growth factor 1 (IGF1) levels that are inadequately controlled by octreotide or lanreotide (2). Long-term outcomes from clinical studies have demonstrated effective and consistent biochemical control with pasireotide for up to 304 weeks of treatment (3). Moreover, approximately 38% of patients showed significant tumour volume reduction (4). Recently, Coopmans *et al*. (5) reported a significant increase in the T2-weighted magnetic resonance imaging (MRI) signal of adenomas in acromegaly patients treated with pasireotide. This increase was generally found when there was cystic degeneration, necrosis or both, suggesting, according to the authors, an antitumour effect of pasireotide (5). They also described successful dose reduction in two patients because of substantial IGF1 reduction (5).

Herein, we present a retrospective analysis of a subset of patients who received long-term treatment with pasireotide, who maintained their biochemical response and had improved glycaemic control following dose reduction of pasireotide.

## Materials and methods

Data, including growth hormone (GH), IGF1 and tumour parameters, from a prospectively maintained database of patients in clinical studies with pasireotide (parental studies: CSOM230C2305 (6), CSOM230C2402 (7), CSOM230C2413 (8), CSOM230B2219 (9); rollover study: CSOM230B2412) were evaluated. Patients who completed the clinical studies and were judged by the investigator to be benefiting from treatment were enroled in the rollover study and continued treatment with pasireotide in a real-life setting. All studies were approved by the institutional review board/ethics committee of Hospital Universitário Clementino Fraga Filho, and patients provided written informed consent to participate in the parental studies and additionally for the rollover study.

In the parental studies, dose adjustments and efficacy and safety assessments were performed according to each protocol. In the rollover study, these were performed at the investigators’ discretion. During the rollover, in our centre, pasireotide dose was reduced in patients with IGF1 levels in the lower half of the normal range and those with IGF1 levels in the upper half, but glucose control was intended for the latter. There was no specific threshold that indicated dose reduction in these cases; patients were evaluated individually, weighing the risks and benefits of the adjustment. IGF1 was measured by the central laboratory during parental studies using different assays described in the respective studies, and during the rollover study, it was measured locally using a chemiluminescence assay kit (IMMULITE; DPC – Diagnostic Products Corp., Inc., Los Angeles, CA, USA), therefore we reported IGF1 levels as a relation to the upper limit of normal (×ULN). For this analysis, biochemical control was considered as IGF1 levels ≤ 1.0 × ULN. Numerical variables are expressed as median (range) and were compared by Mann–Whitney test. A *P* value of <0.05 was considered significant.

## Results

Of 50 patients treated with pasireotide at our centre, 27 had IGF1 control (IGF1 ≤ 1.0 ULN) with pasireotide, and in 20 of these, dose reduction was possible. In these 20 patients, the median age at diagnosis was 39 (17−67) years and the male-to-female ratio was balanced (Table 1). The median IGF1 concentration before starting pasireotide treatment was 2.4 (1.3−5.2) × ULN. Eight patients were diabetic at baseline, defined by baseline fasting glucose ≥ 126 mg/dL, glycated haemoglobin (HbA1c) ≥ 6.5% or receiving medication for hyperglycaemia. Nine patients had undergone transsphenoidal surgery before pasireotide treatment. Most patients had been treated with first-generation SRLs and/or cabergoline without achieving biochemical control.

**Table 1:** Patient demographics at baseline.

Patient number	Age at diagnosis, years	Gender	Diabetic at baseline^a^	Prior surgery	Prior radiotherapy	Treatment prior to pasireotide	IGF1 prior to starting pasireotide, × ULN	Completed clinical trial
**1**	**38**	**M**	**N**	**Y**	**N**	**Octreotide LAR + cabergoline**	**1.9**	**CSOM230C2402 (7)**
**2**	**54**	**F**	**N**	**N**	N	**Octreotide LAR**	**3.0**	**CSOM230C2402 (7)**
**3**	**33**	**M**	**Y**	**N**	N	**Octreotide LAR**	**3.1**	**CSOM230C2402 (7)**
**4**	**25**	**F**	**N**	**Y**	N	**Octreotide LAR + cabergoline**	**1.7**	**CSOM230C2402 (7)**
**5**	**23**	**M**	**Y**	**Y**	N	**Octreotide LAR + cabergoline**	**2.2**	**CSOM230C2402 (7)**
**6**	**42**	**M**	**Y**	**Y**	N	**Octreotide LAR**	**2.3**	**CSOM230C2402 (7)**
**7**	**44**	**M**	**N**	**Y**	N	**Octreotide LAR**	**2.0**	**CSOM230C2305 (6)**
**8**	**48**	**F**	**N**	**Y**	N	**Octreotide LAR + cabergoline**	**2.7**	**CSOM230C2402 (7)**
**9**	**45**	**M**	**N**	**Y**	N	**Octreotide LAR + cabergoline**	**3.5**	**CSOM230B2219 (9)**
10	35	F	Y	Y	N	Octreotide LAR	2.5	CSOM230C2402 (7)
11	20	M	N	Y	N	Octreotide LAR	1.4	CSOM230C2402 (7)
12	34	F	Y	Y	N	Octreotide LAR + cabergoline	1.8	CSOM230C2402 (7)
13	21	M	N	Y	N	Octreotide LAR	1.7	CSOM230C2402 (7)
14	31	F	N	Y	N	Octreotide LAR + cabergoline	1.3	CSOM230C2402 (7)
15	61	F	Y	N	N	–	5.2	CSOM230C2305 (6)
16	49	F	N	Y	N	–	2.4	CSOM230C2305 (6)
17	57	M	Y	Y	N	–	1.6	CSOM230C2305 (6)
18	67	F	Y	N	N	Octreotide LAR + cabergoline	1.4	CSOM230C2413 (8)
19	17	M	N	Y	N	Octreotide LAR + cabergoline	2.2	CSOM230B2219 (9)
20	39	M	N	Y	N	Octreotide LAR + cabergoline	3.4	CSOM230B2219 (9)

Bold indicates late responders (after ≥4 months of treatment).

^a^Defined as baseline glucose ≥ 126 mg/dL, HbA1c ≥ 6.5% or receiving medication for hyperglycaemia.

F, female; LAR, long-acting repeatable; M, male; N, no; Y, yes

**Figure 1:**
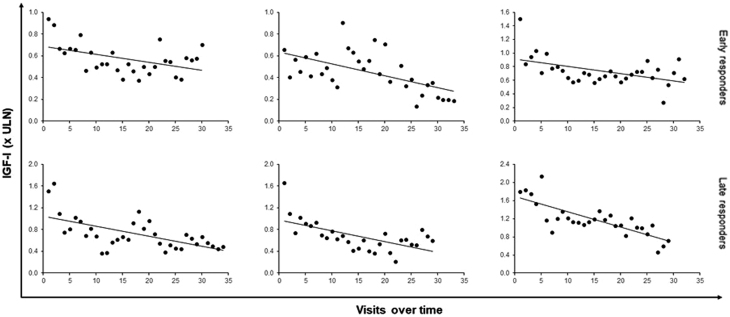
Examples of IGF1 reduction over time in individual patients receiving long-term treatment with pasireotide. Dots show IGF1 levels at each visit over time from the first IGF1 measured after pasireotide initiation to the last IGF1 measured before pasireotide dose reduction. Top graphs correspond to early responders and bottom graphs correspond to late responders. Black lines show the linear trend.

**Figure 2:**
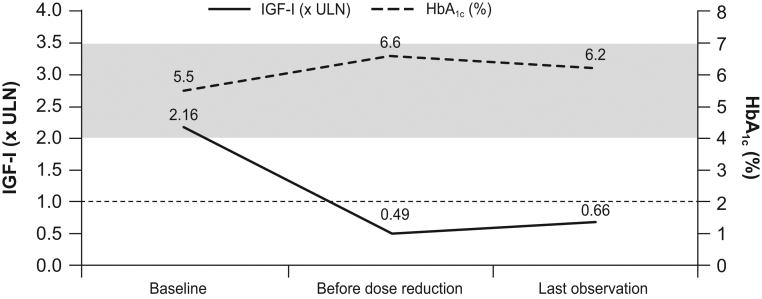
Median IGF1 and HbA1c levels over time in all patients. Dotted horizontal line indicates ULN for IGF1. Dark grey-shaded band indicates control range for HbA1c (4.0–7.0%).

Patients received pasireotide treatment for 6 months to 11 years (median follow-up 9 years) before dose reduction. Starting doses were 40 mg (*n* = 17) or 60 mg (*n* = 3), with 16 patients being ultimately treated with 20 mg and 4 patients with 40 mg. For this analysis, patients were divided into two subsets: long-term responders, who achieved IGF1 ≤ULN after ≥4 months of pasireotide treatment (*n* = 9; median time to IGF1 normalisation 9 (6–54) months), and early responders, who achieved IGF1 ≤ULN within the first 3 months, when the first IGF1 evaluation was performed in most cases (*n* = 11). Most patients showed a progressive reduction in IGF1 levels (Fig. 1), including early responders. Median IGF1 levels decreased from 2.16 × ULN at baseline to 0.49 × ULN before pasireotide reduction (*P* < 0.001) and after dose reduction it increased to 0.66 × ULN (*P* = 0.081 compared with IGF1 values before reduction) (Fig. 2). Early and late responders did not differ in respect to sex, age at diagnosis, impaired glucose metabolism (diabetes, impaired fasting glucose or glucose tolerance), GH and IGF1 levels at diagnosis or before pasireotide treatment and tumour volume at diagnosis.

**Figure 3:**
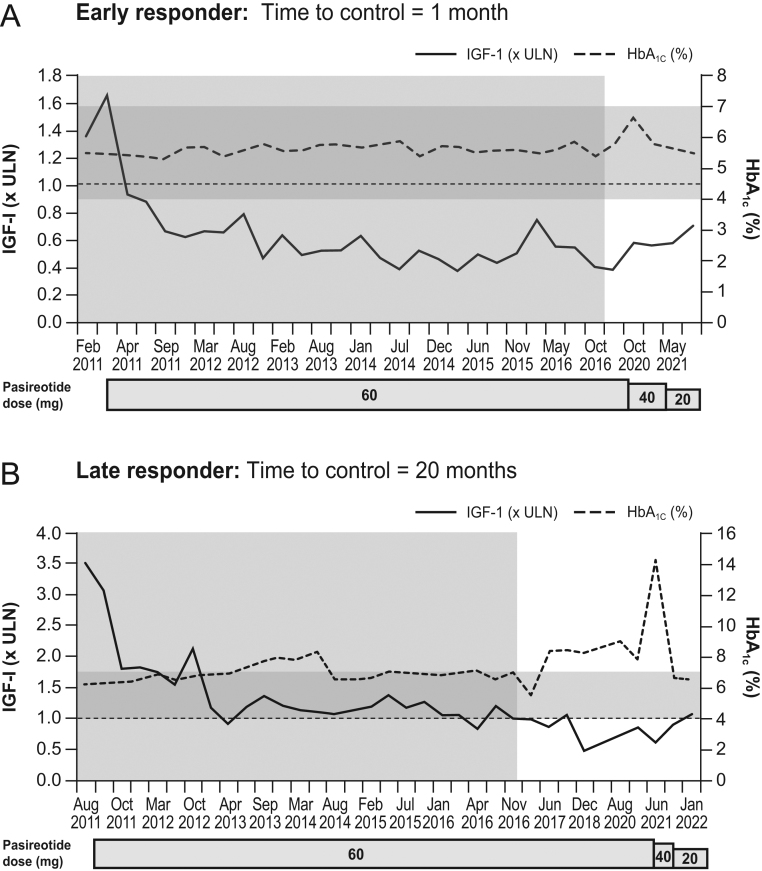
IGF1 and HbA1c levels over time in a patient responding early to pasireotide treatment (A) and a patient responding late to pasireotide treatment (B), both of whom were managed with a reduction in pasireotide dose. Dotted horizontal line indicates ULN for IGF1. Light grey-shaded area indicates length of time the patient was in a parental clinical trial before entering the rollover study. Dark grey-shaded band indicates control range for HbA1c (4–7.0%). Long-term responders achieved IGF1 ≤ ULN ≥4 months after starting pasireotide treatment. Early responders achieved IGF1 ≤ ULN within the first 3 months.

Among early responders, nine patients started on 40 mg and two started on 60 mg. Pasireotide dose was reduced to 20 mg in all patients, and IGF1 remained within normal limits for a median of 39 months (7–120) after dose reduction. Median IGF1 levels were 0.42 (0.27–0.88) and 0.69 (0.18–0.97) × ULN before and after dose reduction, respectively. For example, in patient #13, IGF1 levels were 1.66 × ULN before starting pasireotide and 0.94 × ULN after 1 month of treatment. IGF1 levels progressively decreased, and after 114 months, they were 0.58 × ULN when pasireotide dose was reduced to 40 mg (Fig. 3A). As IGF1 was maintained at the same level, the dose was further reduced to 20 mg, with IGF1 remaining stable (last observed IGF1 after 7 months on 20 mg was 0.70 × ULN). This patient did not display significant hyperglycaemia, except for an isolated HbA1c level of 6.7%. At the last observation, the fasting glucose level was 98 mg/dL and HbA1c was 5.5%.

Among late responders, the starting dose was 40 mg in eight patients, which was increased in seven patients to 60 mg because of inadequate control. One patient started on 60 mg. Owing to a progressive decrease in IGF1 levels, the pasireotide dose could also be reduced, albeit later than for early responders. At last observation, five patients were being treated with pasireotide 20 mg and four with 40 mg. Median IGF1 levels before the last dose reduction were 0.49 (0.21–0.68) × ULN, and after a median of 17 (7–33) months following dose reduction, they were 0.59 (0.45–1.06) × ULN. The patient starting with 60 mg (patient #3) achieved normal IGF1 after 20 months of treatment; afterwards, IGF1 levels fluctuated around the ULN (Fig. 3B). After 87 months of treatment, IGF1 levels started to further decrease, reaching 0.46 × ULN. Meanwhile, glucose levels started to increase, with HbA1c levels peaking at 14.5% at month 117 (baseline levels were 6.2%), despite antidiabetic treatment. So, it was decided to decrease the pasireotide dose to 40 mg after 112 months of treatment, and subsequently to 20 mg after 118 months. After dose reduction, fasting glucose and HbA1c levels progressively decreased. The last observed IGF1, fasting glucose and HbA1c levels under treatment with 20 mg were 1.06 × ULN, 120 mg/dL and 6.5%, respectively.

HbA1c levels in most patients remained stable over time, and glucose fluctuations stabilised or decreased following pasireotide dose reduction. Overall, median glucose levels increased from baseline to immediately before pasireotide dose reduction (100 (86–131) to 129 (95–437) mg/dL; *P* = 0.001), then returned to levels similar to baseline after dose reduction (109 (74–219) mg/dL; *P* = 1.0; Table 2). Median HbA1c levels also increased from baseline to before dose reduction (5.5% (4.5–7.6) to 6.6% (4.2–14.8); *P* < 0.001), then generally decreased but did not reach baseline levels (6.2% (4.2–9.7); *P* = 0.022; Table 2 and Fig. 2).

**Table 2:** Median glucose and HbA1c levels at baseline and before and after dose reduction in all patients (*n* = 20).

	Baseline	Before pasireotide dose reduction	After pasireotide dose reduction	*P* value: baseline vs before pasireotide dose reduction	*P* value: before vs after pasireotide dose reduction	*P* value: baseline vs after pasireotide dose reduction
Median glucose, mg/dL (range)	100 (86–131)	129 (95–437)	109 (74–219)	0.001	0.017	1.0
Median HbA1c, % (range)	5.5 (4.5–7.6)	6.6 (4.2–14.8)	6.2 (4.2–9.7)	<0.001	0.086	0.022

There was a significant reduction in median largest tumour diameter (1.3 (0.5–2.6) to 0.7 (0–1.6) cm; *P* = 0.015) in patients with available data before and after pasireotide treatment (at last available assessment; *n* = 11). Tumour reduction was not correlated with IGF1 decrease during pasireotide treatment.

## Discussion

In this retrospective analysis, we showed that pasireotide had prolonged and progressive effects on biochemical control in the treatment of patients with acromegaly. We identified two groups of patients who responded either early (within the first 3 months) or later (after ≥4 months) to treatment, although most patients overall showed a clear tendency toward progressively lower IGF1 levels. This progressive effect allowed us to reduce the pasireotide dose in 20 of 50 patients (40%) treated at our centre.

Successful de-escalation of pasireotide dose has been demonstrated in some case series (10, 11, 12). Akirov *et al.* (10) described pasireotide dose reduction in eight patients for hyperglycaemia, very low IGF1 levels and hair loss. One patient had elevated IGF1 levels after dose reduction, whereas levels remained normal in the other patients (10). Similarly, IGF1 levels showed a mild increase in most of our patients, but all remained within the normal range. Pasireotide dose reduction in a larger series was first described in the extension phase of the PAPE study (13). In this study, the authors described that among 15 patients treated with pasireotide 60 mg in monotherapy, in 10 patients IGF1 levels dropped to the lower half of the normal range, and in these patients, it was possible to reduce pasireotide dose to 40 mg. Out of these 10 patients, in 5 patients, it was possible to further reduce the dose to 20 mg. However, they do not describe dose reduction in patients with IGF1 levels in the upper half of the normal range nor the effects of pasireotide dose reduction on glucose levels (13).

Among the late responders, there were patients that were controlled after a long period of treatment, which was possible due to this progressive IGF1 decrease. Among our patients, most had been operated on and treated with first-generation SRLs and/or cabergoline. Patients were not suitable for another surgical intervention because MRI did not show a clear residual lesion or showed a lesion that could not be resected. Pegvisomant is not available in the public health service in our country, and treatment with first-generation SRL and/or cabergoline would not be effective in these patients. Radiotherapy would be an option; however, we were observing a progressive decrease in IGF1 levels associated with symptom amelioration. For all these reasons, it was decided, in the multidisciplinary team meeting, to observe the patients for a longer period until they were ultimately controlled. So, in patients with a biochemical and clinical response, it may be advisable to wait a longer period before opting for alternative therapy.

As many patients had had surgery and/or medical treatment before receiving pasireotide, evaluation of tumour MRI was limited; some patients had no residual lesions or had lesions that could not be properly measured. This also compromised our evaluation of the T2-weighted signal. Nevertheless, a significant decrease in the largest tumour diameter was seen after treatment with pasireotide in the 11 evaluable patients. Further to the findings of Coopmans *et al.* (5), who showed that an increase in T2-weighted MRI signal in the adenomas of patients treated with pasireotide is associated with cystic degeneration and/or necrosis, we hypothesise that the progressive antisecretory effect of pasireotide described here may be, at least partially, due to this antiproliferative effect. With the reduction of viable tumour load and subsequent decrease in hormone secretion, progressive dose reduction of pasireotide was possible. We did not find a correlation between tumour reduction and IGF1 reduction, probably due to the limited number of patients with tumour reduction information available.

Furthermore, glucose and HbA1c levels decreased after dose reduction, showing that it may be beneficial in some patients for improving glycaemic control while maintaining long-term biochemical response.

One of the early-responder patients was included in an earlier publication describing two patients who over-responded to pasireotide and had the dose reduced (12). These early-responder patients appeared to have increased sensitivity to pasireotide and possibly a higher than necessary initial pasireotide dose. So, it may be recommended to closely observe the initial response of patients and to attempt a dose reduction as soon as feasible, possibly avoiding increases in glucose levels. The mechanisms associated with this early response are unclear, but it can be speculated that such patients may present high expression of somatostatin receptor subtype 5 (SST5). It has been demonstrated that, in patients resistant to first-generation SRL (which was the case in most of our patients), SST5 expression was associated with better response to pasireotide (15). Unfortunately, there was no available material for evaluation of somatostatin receptors (SST) expression in our series of patients. All the parameters evaluated (clinical, biochemical and radiographical) were not different between early and late responders.

Altogether, our findings suggest that pasireotide dose reduction should be considered in biochemically controlled patients, especially those who have IGF1 levels in the lower half of the normal range. However, as IGF1 levels remained stable in most patients following dose reduction, it should also be considered in patients with levels in the upper half of the normal range who have hyperglycaemia.

## Declaration of interest

MG has received speaker fees from Recordati Rare Diseases, Ipsen, Crinetics Pharmaceuticals, and Novo Nordisk and attended advisory boards for Novo Nordisk, Recordati Rare Diseases and Crinetics Pharmaceuticals. NVM has received speaker fees from Novartis and Ipsen and attended advisory boards for Crinetics Pharmaceuticals. LEW has received speaker fees from Novartis and Ipsen and participated in advisory boards for Crinetics Pharmaceuticals.

## Funding

The parent studies and rollover study were funded by Novartis Pharma AG; however, as of 12 July 2019, pasireotide is an asset of Recordati. Financial support for medical editorial assistance was provided by Recordati.

## Author contribution statement

All authors contributed to data interpretation, writing and reviewing of the manuscript, including the decision to submit the manuscript for publication, and can confirm the accuracy and completeness of the data.
